# The complete chloroplast genome sequence of *Pittosporum kerrii*: the first Pittosporaceae plastome

**DOI:** 10.1080/23802359.2019.1688712

**Published:** 2019-11-12

**Authors:** Yi Wang, Chuan Zhao, Yunqing Li

**Affiliations:** Laboratory of Forest Plant Cultivation and Utilization, Yunnan Academy of Forestry, Kunming, People’s Republic of China

**Keywords:** *Pittosporum kerrii*, chloroplast, Illumina sequencing, phylogenetic analysis

## Abstract

The first complete chloroplast genome (cpDNA) sequence of *Pittosporum kerrii* was determined from Illumina HiSeq pair-end sequencing data in this study. The cpDNA is 153,581 bp in length, contains a large single-copy region (LSC) of 84,940 bp and a small single-copy region (SSC) of 18,741 bp, which were separated by a pair of inverted repeats (IR) regions of 24,950 bp. The genome contains 132 genes, including 87 protein-coding genes, 8 ribosomal RNA genes, and 37 transfer RNA genes. The overall GC content of the whole genome is 38.3%, and the corresponding values of the LSC, SSC, and IR regions are 36.5, 32.5, and 43.3%, respectively. Further, phylogenomic analysis showed that *P. kerrii* clustered in a unique clade in order Apiales.

*Pittosporum kerrii* is the species of the genus *Pittosporum* within the family Pittosporaceae (Yang et al. 2016). It distributes in Yunnan of China, Thailand, Myanmar (Wu et al. 2018). *Pittosporum kerrii* is famous ethnomedicines in southwestern China (Luo et al. 2014). Several chemical constituents including phenylpropanoid, sterol, and isobenzofuran lactone derivatives have been isolated from the roots (Yang et al. 2016) and bark (Zhang et al. 2015) of *P. kerrii*. The extract of *P. kerrii* also showed several bioactive, such as antitumor, antibacterial, and anti-HIV (Éparvier et al. 2007). Therefore, *P. kerrii* has huge potential medicinal value (Luo et al. 2018). However, there have been no genomic studies on *P. kerrii*.

Herein, we reported and characterized the complete *P. kerrii* plastid genome (MN539266). One *P. kerrii* individual (specimen number: 5309270711) was collected from Cangyuan, Yunnan Province of China (23°12′23″N, 99°12′39″ E). The specimen is stored at Yunnan Academy of Forestry Herbarium, Kunming, China and the accession number is YAFM20180412. DNA was extracted from its fresh leaves using DNA Plantzol Reagent (Invitrogen, Carlsbad, CA, USA).

Paired-end reads were sequenced by using the Illumina HiSeq system (Illumina, San Diego, CA, USA). In total, about 24.7 million high-quality clean reads were generated with adaptors trimmed. Aligning, assembly, and annotation were conducted by CLC de novo assembler (CLC Bio, Aarhus, Denmark), BLAST, GeSeq (Tillich et al. 2017), and GENEIOUS version 11.0.5 (Biomatters Ltd, Auckland, New Zealand). To confirm the phylogenetic position of *P. kerrii*, other seven species of Order *Apiales* from NCBI were aligned using MAFFT version 7 (Katoh and Standley 2013). The Auto algorithm in the MAFFT alignment software was used to align the eight complete genome sequences and the G-INS-i algorithm was used to align the partial complex sequecnces and maximum likelihood (ML) bootstrap analysis was conducted using RAxML (Stamatakis 2006); bootstrap probability values were calculated from 1000 replicates. *Viburnum japonicum* (MH036493) and *Dipsacus asper* (MH074864) were served as the out-group.

The complete *P. kerrii* plastid genome is a circular DNA molecule with the length of 153,581 bp, contains a large single-copy region (LSC) of 84,940 bp, and a small single-copy region (SSC) of 18,741 bp, which were separated by a pair of inverted repeats (IR) regions of 24,950 bp. The overall GC content of the whole genome is 38.3%, and the corresponding values of the LSC, SSC, and IR regions are 36.5, 32.5, and 43.3%, respectively. The plastid genome contained 132 genes, including 87 protein-coding genes, 8 ribosomal RNA genes, and 37 transfer RNA genes. Phylogenetic analysis showed that *P. kerrii* clustered in a unique clade in Order *Apiales*, which indicated the phylogenesis classification of *P. kerrii* (Figure 1). The determination of the complete plastid genome sequences provided new molecular data to illuminate the *Apiales* evolution.

**Figure 1.**
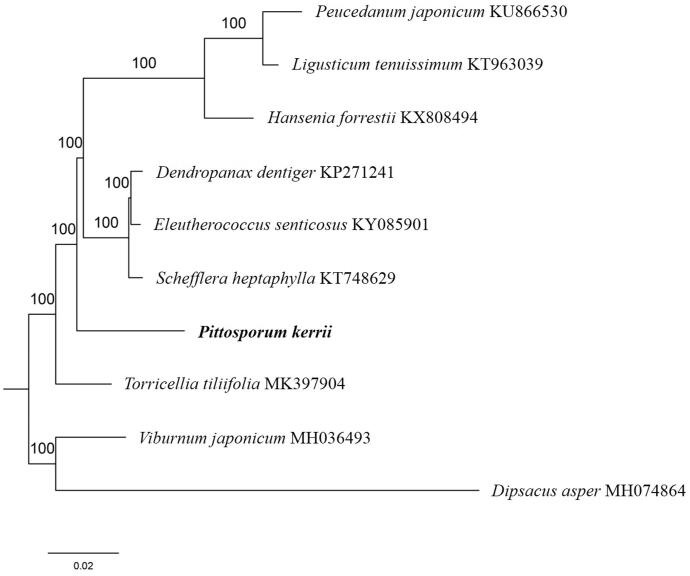
The maximum-likelihood tree based on the eight chloroplast genomes of *Apiales*. The bootstrap value based on 1000 replicates is shown on each node.
